# Annotations

**Published:** 1896-07-25

**Authors:** 


### Annotations.

### Metropolitan Workhouse Children.

A circular has been issued by the Secretary of the Howard Association criticising the report of the Poor Law Schools Committee, the conclusions of which are stated to be not supported by the evidence. "Witnesses have been misrepresented. What is favourable to the schools and the management of the Local Government Board and guardians has been suppressed." This is a very strong statement, and if it can be supported it may be necessary to reconsider the conclusions which have been arrived at. It will be remembered that it was recommended that the Poor Law children of the metropolis should be transferred from the care of the guardians and the Local Government Board to fresh bodies and authorities; and in regard to this the circular we have received points out that so far as boarding out is concerned, which seems to be the object chiefly aimed at by those who advocate the change, great difficulties are likely to be experienced in consequence of two separate bodies being engaged in boarding our children. There would have to be two separate sets of inspectors and of visiting committees, and even if the same committees were authorised to receive children both from the provincial unions and the metropolitan body, the committees would be under the control of two bodies, which would certainly result in friction. What the Howard Association recommends, then, is that a children's department should be formed under the Local Government Board, which would deal with the children of both the metropolitan and provincial unions. The great advantage of this would be that the children would still be under a Government department, to the dictates of which the visiting committees would not hesitate to submit, while, on the other hand, it being a separate department its work would not be crowded out by the multitude of urgent duties which are at times thrown upon the Local Government Board.

### Insusceptibility to Vaccination.

In his annual report, just published, Dr. Thorne Thorne raises a question of much importance, both from a scientific and a practical point of view. It appears that in 1892 no fewer than 1,983 children were certified as insusceptible of vaccination. Now the medical department of the Local Government Board does not believe that any children are insusceptible of vaccination; and among other reasons it bases its opinions upon the fact that the department, having been directly responsible for a large number of vaccinations extending over several years, has never yet found a child who proved insusceptible to vaccination when operated upon with reasonable skill. Is the department right in its judgment, or are the hundreds of medical men who so constantly certify insusceptibility? The bacteriologist, we feel sure, will hold that the department is right. The facts are very plain. Vaccine lymph consists of definite organisms, which being planted in the suitable soil of human tissues, begin to reproduce themselves exactly as wheat or any other seed or spore does in suitable soil. That some soils grow wheat better than others is common experience. But that any soil in the world is such as to be incapable of causing a grain of wheat to sprout and reproduce itself in some form is a proposition which science cannot accept. Just as little can we believe that human tissues will refuse to reproduce vaccine lymph in the form of a more or less normal pock, when good lymph is inserted with competent care and skill. The medical department might very well issue specially reasoned instructions on this point.

### Hospital Mortuaries.

Nothing is so galling to the friends as any sign of irreverence to, or disrespect in the treatment of, their dead, and nothing is so likely to bring forth angry comments from a jury as the sights and smells of an ill-kept mortuary. Yet the dead-house is a sort of no man's land. The medical staff have already seen the last of their patient, the nurses have performed their last kind offices, the porters have carried him down, and their duty is done. Poor fellow, he lies literally on the shelf; and as for the undertaker's men, they are imagined not to care; so that unless a steward or some one in authority goes that way, or unless a coroner's jury bent on viewing a body take cognisance of its surroundings and complain about them, the hospital mortuary is apt to be left to the care of hirelings of low degree, and to be neglected. A complaint has recently been made in regard to the management of the mortuary attached to St. Thomas's Hospital. A jury visited this place to view two bodies. To do this the coffin lids had to be partially removed, and the stench was sickening. It was also noticed that blood was on the floor. The jury were so strongly impressed with the experience they had gone through that, at their unanimously expressed wish, the foreman wrote to the authorities at the hospital, drawing their attention to the evils which had been complained of, and suggesting that covered and partially glazed coffins should be used for bodies which have to be viewed by coroners' juries. To this letter a very satisfactory reply was promptly received from the steward of St. Thomas's Hospital, expressing great regret at what had happened, explaining that the mortuary contained an exceptionally large number of bodies, stating that those responsible for the cleanliness of the place had been "sharply reprimanded" for their neglect, and promising to adopt the suggestion as to the partially glazed coffins in cases on which inquests were to be held. We mention this case partly because we know that the dead-house is very apt not to be inspected as often as other parts of a hospital, and partly because the immediate action taken by the steward shows how ready hospital officials are to rectify any defects in administration which are civilly and intelligibly brought to their notice.

